# Imaging Hallmarks of Sarcoma Progression Via X-ray Computed Tomography: Beholding the Flower of Evil

**DOI:** 10.3390/cancers14205112

**Published:** 2022-10-19

**Authors:** Elena Popova, Sergey Tkachev, Igor Reshetov, Peter Timashev, Ilya Ulasov

**Affiliations:** 1 World-Class Research Centre “Digital Biodesign and Personalized Healthcare”, Sechenov First Moscow State Medical University (Sechenov University), 119991 Moscow, Russia; 2 University Clinical Hospital No. 1, I. M. Sechenov First Moscow State Medical University, Ministry of Health of the Russian Federation (Sechenov University), 119991 Moscow, Russia; 3 Group of Experimental Biotherapy and Diagnostic, Institute for Regenerative Medicine, World-Class Research Centre “Digital Biodesign and Personalized Healthcare”, I.M. Sechenov First Moscow State Medical University (Sechenov University), 119991 Moscow, Russia

**Keywords:** sarcoma, cancer imaging, computed tomography, PET/CT, micro-CT

## Abstract

**Simple Summary:**

Sarcomas represent the largest group of rare solid tumors that arise from mesenchymal stem cells and are a leading cause of cancer death in individuals younger than 20 years of age. There is an immediate need for the development of an algorithm for the early accurate diagnosis of sarcomas due to the high rate of diagnostic inaccuracy, which reaches up to 30%. X-ray computed tomography is a non-invasive imaging technique used to obtain detailed internal images of the human or animal body in clinical practice and preclinical studies. We summarized the main imaging features of soft tissue and bone sarcomas, and noted the development of new molecular markers to reach tumor type-specific imaging. Also, we demonstrated the possibility of the use X-ray computed microtomography for non-destructive 3D visualization of sarcoma progression in preclinical studies. Finding correlations between X-ray computed tomography modalities and the results of the histopathological specimen examination may significantly increase the accuracy of diagnostics, which leads to the initiation of appropriate management in a timely manner and, consequently, to improved outcomes.

**Abstract:**

Sarcomas are a leading cause of cancer death in individuals younger than 20 years of age and represent the largest group of rare solid tumors. To date, more than 100 morphological subtypes of sarcomas have been described, among which epidemiology, clinical features, management, and prognosis differ significantly. Delays and errors in the diagnosis of sarcomas limit the number of effective therapeutic modalities and catastrophically worsen the prognosis. Therefore, the development of an algorithm for the early accurate diagnosis of sarcomas seems to be as important as the development of novel therapeutic advances. This literature review aims to summarize the results of recent investigations regarding the imaging of sarcoma progression based on the use of X-ray computed tomography (CT) in preclinical studies and in current clinical practice through the lens of cancer hallmarks. We attempted to summarize the main CT imaging features of soft-tissue and bone sarcomas. We noted the development of new molecular markers with high specificity to antibodies and chemokines, which are expressed in particular sarcoma subtypes to reach tumor type-specific imaging. We demonstrate the possibility of the use of X-ray computed microtomography (micro-CT) for non-destructive 3D visualization of solid tumors by increasing the visibility of soft tissues with X-ray scattering agents. Based on the results of recent studies, we hypothesize that micro-CT enables the visualization of neovascularization and stroma formation in sarcomas at high-resolution in vivo and ex vivo, including the novel techniques of whole-block and whole-tissue imaging. Finding correlations between CT, PET/CT, and micro-CT imaging features, the results of the histopathological specimen examination and clinical outcomes may significantly increase the accuracy of soft-tissue and bone tumor diagnostics, which leads to the initiation of appropriate histotype-specific management in a timely manner and, consequently, to improved outcomes.

## 1. Introduction

Sarcomas represent the largest group of rare solid tumors that arise from mesenchymal stem cells, although among subtypes, the ultimate cells of origin remain unclear [1,2]. Currently, more than 100 morphological subtypes of sarcomas have been described, among which epidemiology, clinical features, management, prognosis, and outcomes may differ significantly [3,4]. Sarcomas account for nearly 20% of all pediatric cancers and less than 1% of adult cancers [5,6]. Matching the formal definition of a rare tumor, the incidence of sarcomas is 5.6 cases per 100,000 per year in Europe, with 27,908 new diagnoses per year, of which 84% were soft-tissue sarcomas (STS) and 15% were bone sarcomas [7,8]. Despite the relative rarity of diagnosis, sarcomas are a leading cause of cancer death in individuals younger than 20 years of age [9]. In the era of modern therapeutic advances for sarcomas’ management that resulted in improved outcomes, statistics remain devastating. The Eurocare-5 survival study has shown a 60% overall 5-year survival rate for STS and about 50% for bone sarcomas [10,11]. Overall, approximately 10–20% of STS recur locally, and 25% of patients with STS develop distant metastatic disease after successful treatment of their primary tumor [12,13]. In 70 to 80% of cases, metastatic disease was found in the lungs [14]. Up to one-half of patients with high-grade STS die from tumor-related disease [15].

Undifferentiated pleomorphic sarcoma (UPS), liposarcoma, and leiomyosarcoma are the most common STS subtypes, comprising about 65% of STS cases among adults [4]. In children, rhabdomyosarcoma is the most common type, which accounts for approximately one-half of all STSs in this age group [16]. Osteosarcoma and the Ewing sarcoma family of tumors, e.g., Ewing sarcoma of bone, extraosseous Ewing tumors, and peripheral neuroepithelioma (PNET), represent the three most common pediatric sarcomas [17]. Among cases of bone sarcomas in adults between 30 and 60 years of age, chondrosarcoma is the most frequent subtype [18].

Aggressiveness, histopathologic heterogeneity, and the presence of therapeutic resistance in sarcomas are daunting. An open incisional or core needle biopsy and subsequent histologic examination are essential for diagnosis, but can be challenging, even for well-qualified and experienced surgeons and pathologists [19,20,21]. The diagnostic process’s relative rarity and technological complexity may explain a rate of diagnostic inaccuracy of up to 30% [22] Delay in diagnosis of sarcomas is also common, e.g., the SURVSARC study shows the diagnostic interval length was ≥1 month in 55% of patients, and ≥3 months in 28% of patients [23]. Sarcomas do not have any specific clinical signs, so most primary care doctors do not suspect the presence of a malignant solid tumor: the most common symptom is the occurrence of a large, painless mass in the extremities or trunk wall [24], and paraneoplastic symptoms such as fever may occur very rarely [25]. Delayed diagnosis catastrophically worsens the prognosis and limits the number of available therapeutic interventions [26,27]. The development of an algorithm for the early accurate diagnosis of sarcomas seems to be as important as the development of novel therapeutic advances.

**Figure 1:**
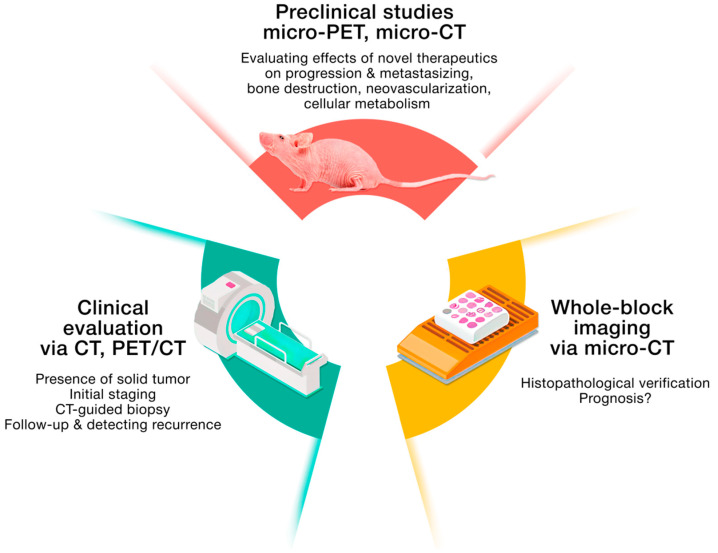
Current imaging techniques and their objectives in sarcoma diagnostics and preclinical studies.

The combination of microscopic morphology evaluation, immunohistochemistry, and molecular diagnostics may improve diagnosis accuracy and timeliness, allowing for the initiation of appropriate histotype-specific management as soon as possible, particularly in very rare tumors such as those represented by the diverse Ewing-like family of tumors [28,29]. However, before biopsy and histological examination of tissue samples, the patient undergoes diagnostic imaging, which remains a crucial step towards accurate diagnosis, effective treatment, and, correspondingly, towards better outcomes. Combined in the correct sequence, plane radiography, ultrasonography, magnetic resonance imaging (MRI), X-ray computed tomography, and positron emission tomography-CT (PET/CT) are essential tools for initial diagnostic workup, staging, prognostication, grading, establishing the presence or absence of metastatic disease, and surgical planning. Moreover, functional imaging (e.g., [^18^F]FDG PET/CT) is necessary to evaluate patient response to treatment [19,20]. As pathologists, radiologists also face many challenges during sarcoma diagnosis, and specialists need to join forces not only in discussion, but at the patient’s bedside, to minimize diagnostic discrepancies [30,31]. Translation scientists enter the scene or, more likely, the battlefield, where they carry out in vitro, ex vivo, and in vivo preclinical studies, estimating the efficacy of new therapeutic strategies via a wide spectrum of laboratory methods, including confocal laser scanning microscopy (CLSM), X-ray computed microtomography (micro-CT), searching for histopathological correlations of sarcomas with imaging findings, and even using artificial intelligence. We summarized the findings of clinical and preclinical imaging studies on soft-tissue and bone sarcomas through the lens of cancer hallmarks, finding correlations with other approaches and techniques [32]. In Figure 1, we briefly listed current imaging techniques and their objectives in sarcoma diagnostics and preclinical studies.

## 2. Clinical Imaging, or Seeing Many Things at Once

Tumor cell proliferation is fueled by sustaining growth signaling, evading growth suppressors, avoiding immune destruction, and resistance to apoptosis [33,34]. Invisible interactions between the elements of the human genome, proteins, and cells can result in apparent clinical imaging signs, such as increasing tissue masses, morphological alterations, and the presence of tumor necrosis or destruction of bone or cartilage tissues. In Table 1, we attempted to summarize the main imaging features of STSs and bone sarcomas. Essentially, this summary cannot substitute for the qualified assessment of imaging data by the radiologist but can be a reminder for clinicians who may occasionally face a suspected relatively rare tumor during the patients’ examination for another reason.

Plane radiographs are commonly used to begin the initial evaluation of newly appearing lesions at the extremities, which are a common site for STSs and bone sarcomas [24,44]. The sequence of using diagnostic methods can significantly affect the correctness of the primary evaluation, especially for malignant bone tumors [45]. Radiography is a cheap, simple, and fast procedure, and it allows clinicians to exclude the presence of injuries and to ascertain the type of lesion (the soft-tissue tumor or lesion that originated from the bone). Plain radiographs were strongly recommended as the first step in the evaluation of a possible bone tumor by the Musculoskeletal Tumor Society and the American Academy of Orthopedics [46]. Then, depending on the patients’ anamnesis and the suspected type of the lesion, the guidelines recommended MRI (for the suspected soft-tissue tumor) and/or CT (for the suspected bone tumor) as an essential part of the initial workup [19,20].

Depending on the availability of health services and specific contraindications, MRI and CT are applied together for the initial and secondary locoregional staging of STSs and bone sarcomas. MRI provides better soft-tissue visualization, and, vice versa, CT provides better visualization of the bony tissues [47]. Certain features on CT are considered significant markers for malignant behavior of the soft-tissue tumor lesion, including the relationship of a soft-tissue tumor to the bones, e.g., osseous involvement (cortical destruction, endosteal and periosteal reaction), the presence of calcification, necrosis, and hypervascularity [47]. CT also allows accurate assessment of lesion vascularity. In a comparison with vascular MRI, CT angiography with three-dimensional reconstruction was found equivalent to MR imaging in its ability to demonstrate neurovascular involvement [37,48]. A CT examination of the chest, to detect metastatic lesions in the lungs, is an essential part of the initial preoperative staging of patients with sarcomas [19,20].

Radiomics is a rapidly developing area to improve the accuracy of the current diagnostic tools [49]. There is growing evidence regarding quantitative MRI as a promising method in the investigation of the radiology–pathology correlations in STSs, providing a “virtual biopsy” to minimize diagnostic discrepancies [50,51]. Studies revealed a possibility of using non-contrasted CT texture analysis to access angiogenesis and predict survival via kurtosis estimation [52,53]. Tian, Hayano and colleagues also used contrast-enhanced CT texture analysis in the assessment of response to neoadjuvant therapy in STS [54]. To date, artificial intelligence (AI) and deep machine learning give us a chance to conduct multimodality imaging via combining CT, PET/CT, and MRI imaging data. The complex approach may increase the accuracy of the diagnostics, as has been shown in recent studies regarding prediction [55]. The recent systematic review analyzed 49 papers regarding CT and MRI radiomics of STSs and bone sarcomas. Gitto and colleagues emphasized the necessity of clinical validation and assessed the reproducibility of the radiomics studies’ results by using various CT and MRI scan datasets, produced by the different scanners in the independent hospitals and centers, and combining different strategies of machine learning model validation [56].

## 3. PET Imaging: A Tool for Revealing and Deregulating Cellular Metabolism and Overcoming the Avoidance of Immune Destruction

Malignant tissues tend to have high metabolic activity and accumulate nutritive substances, such as glucose or amino acids, to a greater degree compared to most normal soft tissues [57]. PET/CT functional imaging permits visualization of one of the cancer hallmarks, which is deregulated cellular metabolism [33]. We can directly observe the Warburg effect via specific radiotracers, e.g., 2-[^18^F]Fluoro-2-deoxy-D-glucose ([^18^F]FDG), and 3′-Deoxy-3′-[^18^F]fluorothymidine ([^18^F]FLT). According to the last clinical guidelines, [^18^F]FDG PET/CT is not recommended for initial local workup of soft-tissue tumors and bone tumors [18,19,20,58], but has an important role in the detection of local recurrence and metastasis spreading. Most sarcomas are FDG-avid, but uptake rates are variable among the histological subtypes and grades of the tumor, and the maximum level of the standardized uptake value (SUV_max_) does not always indicate the grade of a particular sarcoma’s subtype. Finding radiology–histopathology correlations is difficult due to sarcoma heterogeneity and rarity. For example, Hack and colleagues have found that high SUV_max_ predicted worse short-term and long-term outcomes in STS and in Ewing sarcoma family tumors. Conversely, in bone sarcomas, no such correlation was found, but the outcome was strongly related to bone tumor volumes [59]. Low SUV was found in tumors of grades 1-3, implying that a low-tumor SUV does not rule out the presence of a high-grade sarcoma [60]. Nonetheless, studies conclude that high [^18^F]FDG uptake is most likely due to high-grade sarcoma [61]. The accumulated data enable conducting systematic reviews and meta-analyses to assess the prognostic value of [^18^F]FDG PET among the most common sarcoma subtypes, but they have serious limitations, such as significant heterogeneity among the studies. It may be cautiously claimed that [^18^F]FDG-PET can efficiently help to differentiate between benign and malignant bone and soft-tissue tumors [62]. In addition, it has been investigated in Ewing sarcoma that [^18^F]FDG PET/CT is sensitive and accurate in diagnosing, staging, and detecting recurrence compared with non-PET imaging [63], and can predict post-treatment progression-free survival in young patients [64].

Of great interest are current investigations regarding new radiotracers, such as Gallium 68-fibroblast activation protein ([^68^Ga]-FAPI) [61]. Kessler and colleagues’ studies claimed an association between tumoral [^68^Ga]-FAPI PET uptake intensity and histopathologic FAP expression in sarcoma patients [65], but non-tumor specific [^68^Ga]-FAPI uptake in degenerative lesions, muscle, head-and-neck, scarring, mammary glands, or uterus was observed [66].

In preclinical studies, non-invasive PET/CT imaging can serve multiple purposes. Researchers use small animal [^18^F]FDG PET/CT imagers to detect sarcoma growth and development, including metastatic lesions, in mice in vivo [67,68,69,70,71,72]. Other radiotracers, such as the ligands for σ2-receptors, were tested in a murine mammary sarcoma model [73]. Another example is that PET/CT allows evaluation of the effect of new management approaches in rodent models, such as arterial embolization via chitosan micro-hydrogels to reduce the size of locally advanced hindlimb sarcoma [74]. Huang and colleagues reported the successful construction of an anti-human PD-L1 antibody with iodine-124 labeling for noninvasive detection of PD-L1 expression in an osteosarcoma mouse model (human OS-732 cells were inoculated); therefore, this study lays the foundation for further investigations regarding noninvasive osteosarcoma diagnostics and targeted therapy in patients with high levels of PD-L1 receptor expression [75]. The same approach was shown one year later with sarcoma cell homografts in humanized mice [76]. Karkare et al. presented their results regarding targeting insulin growth factor receptor type 2 (IGF2R) by microSPECT/CT imaging in osteosarcoma patient-derived xenografts (PDX) in mouse models and in canine osteosarcoma tumors, which showed selective uptake of the radiolabeled IGF2R-specific antibody. In vivo, radioimmunotherapy with Lutetium-177 antibody significantly slowed tumor growth in both a standard cell line and a patient-derived xenograft (PDX) tumor line. Furthermore, no local or systemic toxicity was observed [77]. The combination of PET/CT and micro-CT imaging was used by Guan et al. to estimate the accuracy of CXCR4-based fluorescent detection of the primary and metastatic sites in human osteosarcoma xenograft models in mice [78]. O’Neill and colleagues constructed the ^64^Cu-labeled anti-CD99 antibody to visualize Ewing sarcoma xenograft tumors and their micrometastases; results were compared with conventional MRI and [^18^F]FDG-PET imaging. Micrometastases that were not detected with [^18^F]FDG–PET were readily visualized with the ^64^Cu-labeled anti-CD99 antibody [79].

## 4. Bone Destruction as a Result of Sarcomas’ Progression and Metastasis

To date, most sarcomas’ modeling studies, in which scientists may apply PET/CT, CT, and micro-CT imaging techniques, are related to bone sarcomas [80]. We can do this through the injection of a concentrated suspension of animal (murine, rat allograft cells) or human (xenograft) tumor cells, either in close contact with the bone or into the bone’ medullary cavity (such as into the femur or tibia) [81]. Models created by injecting established cell lines from the respective tumor types into orthotopic sites are more easily induced than genetically engineered or PDX models [68,82,83,84,85,86,87,88,89,90,91,92,93]. 

Computed X-ray micro-tomography (micro-CT) is an imaging technique enabling 3D non-destructive tissue visualization with a minimum voxel size of 1–5 μm^3^ [94], providing an opportunity to study tumors in their native state as well as their original spatial interactions with the microenvironment [95]. Using micro-CT, we can visualize key changes in bone microarchitecture associated with tumor development in live animals and, moreover, compare them among different osteosarcoma models and quantify these changes via bone volume, and cortical and trabecular bone measurements, such as trabecular number and trabecular thickness [84,85,96,97,98]. We can also study the early stages of tumor growth before there is a palpable tumor mass, determine the type of bone lesion (osteoblastic or osteolytic), and the animal’s apparent metastatic sites [81]. As demonstrated in Heymannan and colleagues’ study of zoledronic acid in combination with ifosfamide in rat osteosarcoma, micro-CT allows for the measurement of a treatment’s influence on bone remodeling in vivo [87].

## 5. Untrodden Path: Vasculature Access in Sarcomas

A blood vessel formation, or neovascularization, which provides tumor growth and allows metastatic spread, is comprised of angiogenesis, vasculogenesis, and vasculogenic mimicry [33,99]. Angiogenesis results in newly formed vessels branching from pre-existing ones, and this process is controlled by basic fibroblast growth factor (bFGF), platelet-derived growth factor (PDGF), and the VEGF-A/VEGFR-2 signaling pathway [100,101]. Vasculogenesis is the formation of blood vessels from the de novo generation of endothelial cells, induced mostly by vascular endothelial-derived growth factor (VEGF) [99]. In vasculogenic mimicry, dedifferentiated “endothelial-like” tumor cells form channels that connect with the existing vasculature [102]. The most common types of neovascularization differ between cancers and may be associated with prognosis [103,104]. The use of targeted imaging of neovascularization should significantly improve the implementation of new therapeutic approaches.

Confocal laser scanning microscopy (CLSM) enables the in vivo or ex vivo examination of tumor-associated progression, neovascularization, and inflammation, and has been used for studying sarcoma biology and preclinical investigations of novel therapies [105,106,107]. However, this method is not always possible to apply due to the substantial thickness of the solid tumor samples, the inhomogeneous absorption of light by various tissues, which in sarcomas are extremely heterogeneous, and the need for the special preliminary preparation of samples to make them permeable to the laser beam. Micro-CT imaging techniques permit the visualization and quantification of the vasculature and organ structure in disease models, both in vivo and ex vivo [108,109]. We may assess the tumor vascular bed structure, model blood flow, analyze the vascular network [110], determine the type of neovascularization, and estimate how the new therapies can modulate neovascularization. The influence of particular therapeutic interventions on the neovascularization and quantification of changed vessel parameters via micro-CT was shown in studies regarding breast cancer models [111,112], uveal melanoma [113], and lung cancer [114]. Interestingly, optical coherence tomography (OCT) angiography was also used as a tool for imaging vascular patterning of subcutaneous mouse fibrosarcomas. Combined with conventional immunohistochemistry staining, OCT revealed that expressed VEGF isoform is related to changes in vascular networks and the thickness of vessels [115]. Furthermore, blood flow contributions to metastatic lesions occurring in a given organ were estimated using micro-CT in a hepatic colorectal metastasis mouse model [116]. It has been shown that micro-CT angiography can clearly demonstrate the morphologic changes of vessels and their relationships with tumors [117]. Compared with other techniques for tumor vessel morphologic evaluation and measurement of vessel content, micro-CT has several additional features. It provides the opportunity to study the three-dimensional structures involved in tumor angiogenesis, such as vascular connectivity, angles, and branches. In preclinical studies, vessel segmentation as a tool for assessment was shown to be a simple and effective method.

In the last 15 years, the development of digital pathology has enabled new approaches for three-dimensional (3D) reconstruction of tumors using whole-slide images (WSI). 3D reconstructions are formed computationally based on images of serial tissue sections. Unfortunately, this method does not allow one to avoid the disadvantages of classical histological examination in the form of unavoidable damage to the sample during the section [118]. Recent studies have shown the efficacy of histological evaluation in breast, lung, colon, gastric, and thyroid cancer using whole-block imaging (WBI) via micro-CT [119,120,121,122,123]. The role of whole-tissue imaging can be increased due to the necessity for accurate evaluation of margins of surgical resection, initial treatment response, and response to neoadjuvant chemotherapy, and would be considered as a helping hand for pathologists. This necessity is easily explained by the abovementioned difficulties in histopathology assessment in sarcomas. Because WBI enables pathologists to review entire FFPE blocks in 3D, it may reduce the workload, e.g., by eliminating the need to recut for deeper layer evaluation. Therefore, we hypothesized that the combination of WSI, WBI, and WTI could provide mutually complementary pathological information for clinical evaluation in sarcomas.

Micro-CT may quantify tumor perfusion and provide an index of vascular complexity, making it a potentially useful addition for clinical detection of vascular normalization in anti-angiogenic trials regarding new therapeutic approaches for sarcomas’ treatment. Kim and colleagues exploit the advantages of in vivo MRI and ex vivo micro-MRI and micro-CT to develop an integrated platform for characterizing angiogenesis at multiple spatial scales in a human breast cancer model [124]. In this study, researchers highlighted that quantifying changes in the tumor vasculature from medical images is often challenging due to limited contrast-to-noise ratio, image artifacts, and spatial resolution limitations [124]. In vivo models help to evade this limitation and invent “relative” imaging biomarkers of abnormal vasculature formation, and the multimodal approach may increase the accuracy of the estimation of tumor neovascularization during the histopathology examination of the patient’s samples via whole-block, whole-slide, and whole-tissue imaging.

**Figure 2:**
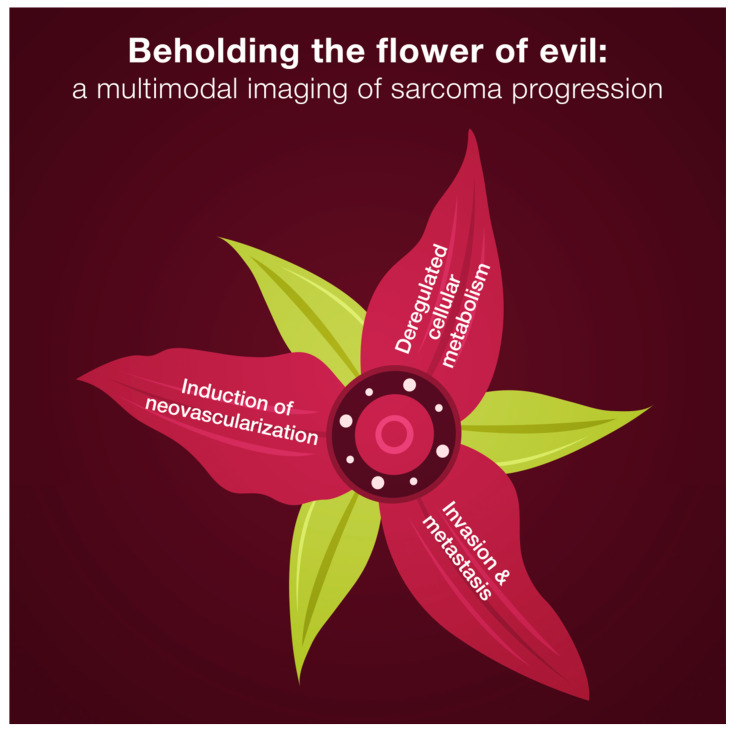
X-ray computed tomography reveals three cancer hallmarks of sarcoma progression.

In Figure 2, we listed cancer hallmarks that we can visualize via CT, micro-CT and micro-PET. Neovascularization, invasion, and metastasizing can be estimated by micro-CT during preclinical studies of novel therapeutic opportunities in murine or rat models. Moreover, these features can be evaluated in patients’ sarcoma samples via the whole-tissue imaging technique. PET/CT functional imaging permits visualization of one of the cancer hallmarks, which is deregulated cellular metabolism.

We noted that there is limited evidence regarding neovascularization imaging in STSs and bone sarcomas via micro-CT ex vivo and in vivo. Using the combination of immunohistochemistry and micro-CT, Darpolor and colleagues showed that vessel density and vessel tortuosity were significantly reduced in tumors with dexamethasone treatment in the rat 9L gliosarcoma model, and CD31-immunoreactive vessels were sparse in treated tumors [125]. Moding et al. studied radiation-induced vascular changes in primary mouse sarcomas, such as an increase in blood flow shortly after radiation treatment, and a decrease in vascular permeability, which were measured by iodine and dextran accumulation. Interestingly, the mean vascular density measured histologically by CD31 staining on day 4 after radiation did not significantly differ between the treated and control tumors, but fractional blood volume, which was assessed via dual-energy micro-CT, was also significantly greater in treated tumors [86]. Immunohistochemistry staining identifies the presence of blood vessels ex vivo, but it does not estimate vascular function, which may change after treatment. Conversely, micro-CT seems to be a suitable method for assessing processes of neovascularization in vivo and ex vivo. Simultaneously, we may investigate the natural course of disease and estimate the effect of different treatment modalities.

**Figure 3:**
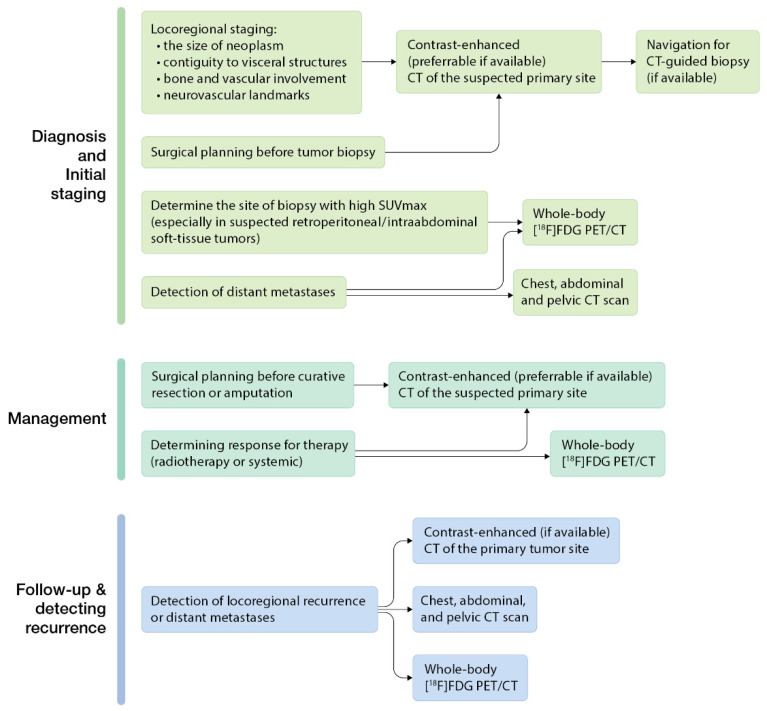
Available CT modalities for sarcoma diagnosis, management, and follow-up (based on The National Comprehensive Cancer Network guidelines [19,20]).

In Figure 3, we briefly summarized available CT modalities for sarcoma diagnosis, management, and follow-up in current clinical practice based on The National Comprehensive Cancer Network guidelines [19,20].

## 6. Conclusions

In this review, we looked at imaging methods for sarcoma progression and summarized the findings of recent studies on imaging sarcoma progression using X-ray computed tomography in preclinical studies and current clinical practice through the lens of cancer hallmarks. The role of CT is limited in the primary evaluation and locoregional staging of STS, but CT scans show the presence of neovascularization and detailed images of the bone involvement and destruction. PET/CT is widely applied in clinical practice for the assessment of sarcoma progression, and SUV_max_ may correlate with prognosis and tumor grade in particular types of STS. The development of radiotracers for molecular imaging via histiotype-specific chemokines and antibodies is a promising solution for increasing diagnostic accuracy. In preclinical studies, PET/CT in vivo imaging provides an assessment of sarcoma progression and metastasizing processes. Micro-CT enables one to estimate changes in bone architecture and neovascularization ex vivo and in vivo, qualitatively and quantitatively, in high spatial resolution; however, we noted that evidence regarding the use of micro-CT in imaging of sarcoma progression is limited. In addition, whole-block imaging seems to be a necessary tool for histopathological specimen examination and may help to avoid diagnostic errors in difficult cases, which are possible due to the variety of histological sarcomas’ subtypes.

Multimodality imaging should help to invent new effective therapeutic modalities which will be precisely targeted for various aspects of sarcoma progression, such as excessive neovascularization and bone destruction. Finding correlations between CT, PET/CT, and micro-CT imaging features, the results of the histopathological specimen examination and clinical outcomes may significantly increase the accuracy of soft-tissue and bone tumor diagnostics, which leads to the initiation of appropriate management in a timely manner, and, consequently, to the improvement of outcomes.

## Figures and Tables

**Table 1:** The main CT features of STSs and bone sarcomas (based on [4,35,36,37,38]).

	CT Features	References
**Chondro-osseous malignant tumors** **Chondrosarcoma** Intramedullary Clear cell	A lesion with calcifications (“ring and arc” or “popcorn” pattern) and aggressive growth features; lytic lesions are also common mixed and sclerotic lesions with visible calcifications (mineralized chondroid matrix present in most cases). Calcifications would be present only in 30% of cases. Heterogeneous pattern that would depend on the proportion of low- and high-grade areas in the lesion.	[39]
**Malignant adipocytic tumors** Well-differentiated liposarcoma: lipoma-like, sclerosing, inflammatory Dedifferentiated liposarcoma Myxoid liposarcoma Pleomorphic liposarcoma Myxoid pleomorphic liposarcoma	The fatty nature of the mass can be proved by the measurement at the field of view (FOV) in Hounsfield units (HU). Fat will show the lowest attenuation of any tissue, and a benign lipoma can be distinguished from a malignant tumor on CT by the uniformly low attenuation (−70 to −130 HU), but it is not possible to reliably differentiate a lipoma from a well-differentiated liposarcoma on CT. However, the presence of a combination of fat and solid components is suggestive of a low-grade liposarcoma. Nonfatty components within an adipocytic tumor should always suggest the possibility of a high-grade liposarcoma. However, it is not always possible to distinguish between the dedifferentiated type and other high-grade liposarcomas. A well-marginated mass of fat attenuation resembling a benign adipocytic tumor, clearly delineating the bony excrescences and adjacent bony cortex; some thickened (more than 2 mm wide) linear or nodular soft-tissue septa during contrast-enhanced CT. Should be suspected if a non-adipocytic component appears in a previously known well-differentiated liposarcoma; retains some of the features of the well-differentiated liposarcoma, while some mass-like areas develop a nonspecific appearance. These areas display tissue attenuation greater than fat on CT scans; calcification or even ossification may be present. Homogeneous or slightly heterogeneous mass that is less attenuating than the surrounding muscle. May occasionally resemble a cyst, due to the lack of fat content, with sharply demarcated margins. It displays attenuation values within the water range (+0 HU). Not distinguishable from other sarcomas because it contains little or no fat.	[38,40]
**Fibroblastic/myofibroblastic malignant tumors** Dermatofibrosarcoma protuberans, fibrosarcomatous Solitary fibrous tumor Inflammatory myofibroblastic tumor Low-grade myofibroblastic sarcoma Superficial CD34-positive fibroblastic tumor Myxoinflammatory fibroblastic sarcomaInfantile fibrosarcoma Solitary fibrous tumor, malignant Fibrosarcoma NOS Myxofibrosarcoma Low grade fibromyxoid sarcoma Sclerosing epithelioid fibrosarcoma)	The lesions have variable attenuation and enhancement on CT scans. Extra-abdominal desmoids are iso- or hypodense relative to the muscle and enhance to +100–110 HU after injection of iodinated contrast material.	[38]
**Malignant tenosynovial giant cell tumor**	A dense soft tissue mass (intra-articular or related to the tendon). CT is useful to detect underlying bone erosions or cysts, contrast-enhanced CT shows hypervascular nature.	[38]
**Malignant vascular tumors** Epithelioid haemangioendothelioma Angiosarcoma **Malignant pericytic (perivascular) tumors** Glomus tumor	Vascular malformations, such as phleboliths and dystrophic calcifications. The involvement of the adjacent joints or bones is possible, such as cortical erosion, periosteal reaction, regional osteopenia, and bony overgrowth. Nonspecific calcified intralesional septa, shown in the contrast enhancement.	[41,42,43]
**Smooth muscle malignant tumors** Inflammatory leiomyosarcoma Leiomyosarcoma	Well-defined, homogeneously enhancing tumors, often associated with fascial edema, with variable signal intensities, central necrosis, and marked peripheral and septal enhancement	[38]
**Skeletal muscle malignant tumors** Embryonal rhabdomyosarcoma Alveolar rhabdomyosarcoma Pleomorphic rhabdomyosarcoma Spindle cell/sclerosing rhabdomyosarcoma Ectomesenchymoma	The majority of STSs have an attenuation value slightly less than that of normal muscle. A nonspecific soft-tissue mass may show local bone invasion, which is seen in about 25% of cases. Bone metastases may occur and are usually lytic and rarely mixed.	[38]
**Peripheral nerve sheath malignant tumors** Malignant peripheral nerve sheath tumor Melanotic malignant nerve sheath tumor Granular cell tumor, malignant Perineurioma	Heterogeneous tumors with necrotic foci. PET/CT: SUV_max_ can assist to separate malignant from benign lesions (especially in case of neurofibromatosis type 1).	[38]
**Malignant tumors of uncertain differentiation** Synovial sarcoma Epithelioid sarcoma: proximal and classic variant Alveolar soft part sarcoma Clear cell sarcoma Desmoplastic small round cell tumor Intimal sarcoma	A soft-tissue mass, which may infiltrate adjacent structures, having a slightly higher density than muscle. Joint invasion and bony involvement, cortical bone erosion, or invasion. Intratumoral calcification or ossification is also more easily seen on CT.Extensive vascular supply led to marked enhancement after injection of contrast medium. A nonspecific soft-tissue mass which may occasionally show punctate calcifications. A nonspecific soft-tissue mass. A nonspecific soft-tissue mass. Multiple omental or serosal soft-tissue masses which have a low attenuation and only moderate homogeneous enhancement, foci of necrosis and calcification. Polypoid intraluminal soft-tissue masses. If not polypoid and no other signs of malignancy are present, the non-enhancing defect may be not distinguishable from thrombus or embolus material.	[38]
**Undifferentiated small round cell sarcomas of bone and soft tissue** Ewing sarcoma Primitive neuroectodermal tumor (PNET)	Tumor with low attenuation. The presence of only focal areas of hypodensity and moderate post-contrast enhancement reflects the differentvascularization pattern. A large, ill-defined mass with a heterogeneous appearance due to extensive cystic degeneration, may be the presence of calcifications. After the injection of iodinated contrast, the tumor has a heterogeneous appearance	[38]
